# Long‐term care issues in a municipality affected by the great East Japan earthquake: A case of Katsurao Village, Fukushima prefecture

**DOI:** 10.1002/ccr3.6268

**Published:** 2022-08-18

**Authors:** Tianchen Zhao, Nobuaki Moriyama, Naomi Ito, Toshiki Abe, Tomohiro Morita, Yoshitaka Nishikawa, Masaharu Tsubokura

**Affiliations:** ^1^ Department of Radiation Health Management Fukushima Medical University School of Medicine Fukushima Japan; ^2^ Department of Public Health Fukushima Medical University School of Medicine Fukushima Japan; ^3^ Department of Internal Medicine Soma Central Hospital Soma Japan; ^4^ Department of Health Informatics Kyoto University School of Public Health Kyoto Japan

**Keywords:** elderly, Fukushima Daiichi nuclear power plant, nuclear emergency, public health, soil contamination

## Abstract

Information regarding the proposed measures addressing long‐term care problems during disasters at a municipal level is scarce. Thus, this study reviewed the long‐term care insurance measures taken in the Katsurao Village after the Fukushima nuclear accident and summarized the measures that municipalities can take against such issues in the future.

## 1 INTRODUCTION

Japan has witnessed a rapid increase in the percentage of those aged 65 years and older. In 2017, 27.7% of the population was aged 65 and older, and in 2016, the percentage of households with individuals 65 years and older was 48.4%.^1^ Subsequently, not only long‐term care insurance premiums, but also the number of older adults requiring long‐term care has increased nationwide.^2^

In the aftermath of the Great East Japan Earthquake and the Fukushima Daiichi Nuclear Power Plant accident, radioactive contamination in surrounding areas exposed residents to radiation, resulting in health problems. Although radiation exposure did not directly affect the health of local residents as reported by international organizations,^3^, ^4^ several diverse and long‐term indirect health effects remain.^5^ Previous studies have suggested that evacuation of local residents from an affected area and the resulting changes in the social environment could affect their health.^6^

In the 12 affected municipalities—mainly in the Soso area, which suffered extensive destruction as a result of the nuclear accident—the need for long‐term care has increased significantly, with this tendency continuing in the future.^7^ Katsurao Village, one of the affected municipalities, faced problems associated with care for older people, necessitating the need to examine the measures to deal with these issues in the affected areas. Although there have been studies on long‐term care for older adults during disasters, such as analyses on health issues of older adults,^8^ caregivers' actions, and insurance data,^6^, ^7^ little information is available regarding the proposed measures addressing long‐term care problems during disasters at a municipal level. Thus, this study aims to review the various long‐term care insurance measures taken in the village and to summarize the measures that municipalities in the affected areas can take against such issues in the future.

## 2 CASE

Katsurao Village is located 30 km northwest of the Fukushima Daiichi Nuclear Power Plant; its pre‐earthquake population was 1567.^9^ Its population has now decreased to approximately 450 people due to evacuation, with 70% of its residents still living outside it. Although the evacuation order was lifted in June 2016 and people have been returning to their villages, 27.7% of Katsurao Village's residents decided not to return according to a survey conducted by a reconstruction agency.^10^ Currently, the number of residents still has not reached the pre‐disaster level.

Because the nuclear accident is a major factor responsible for the increase in the number of people certified as requiring long‐term care, the cost of insurance premiums is also high. Specifically, changes to the living environment have reduced opportunities for farming and other activities, resulting in inactivity in daily life. Family members have been separated due to evacuation, and caregivers are not available. In this situation, the urgent need for long‐term care increased long‐term care insurance premiums among residents aged 65 and older, who paid the highest premiums in Japan from 2018 to 2020. While the national average monthly long‐term care insurance premium was 5869 yen, the monthly premium in Katsurao Village was 9800 yen, about 1.7 times higher. The same tendency was observed in some surrounding municipalities.

In response, Katsurao Village has established a working team to set long‐term care insurance measures. The specific actions taken by the committee can be separated into three categories: analysis of the current situation regarding long‐term care insurance, optimization of long‐term care benefits, and encouraging health promotion and preventive care (Table 1). Through these actions, Katsurao Village aims to curb the cost of long‐term care insurance benefits.

**TABLE 1:** Long‐term care insurance measures taken by a working team in Katsurao Village.

Details of actions	Results of actions
1. Long‐term care services were reviewed in response to the increase in its demand and the number of people certified as requiring long‐term care to control long‐term care insurance benefit costs.	1. The number of people certified as needing long‐term care in Katsurao Village decreased from 155 in FY 2018 to 136 (87.7%) in FY 2021.
2. Inspections of care planning and regular consultations with the community's general support center were carried out, based on which the effectiveness and appropriateness of the long‐term care benefit costs were assessed and optimized.	2. The total cost of long‐term care benefits borne by the Katsurao Village decreased; compared to FY 2018, the cost decreased from 244 million yen to 224 million yen (92.0%) in FY 2021.
3. To raise awareness of health promotion and preventive care, a checklist assessing the risk of long‐term care was utilized.	3. The monthly long‐term care insurance premiums borne by residents in Katsurao Village decreased from 9800 yen in 2018 to 8200 yen (83.7%) in 2021, down from No. 1 to No. 3 in Japan.

First, long‐term care services were reviewed in response to the increase in its demand and the number of people certified as requiring long‐term care to control long‐term care insurance benefit costs. The task force paid particular attention to the analysis and visualization of the current status of the village's long‐term care insurance costs.

Second, inspections of care planning and regular consultations with the community's general support center were carried out, based on which the effectiveness and appropriateness of the long‐term care benefit costs were assessed and optimized.

Third, to raise awareness of health promotion and preventive care, a checklist assessing the risk of long‐term care was utilized. This checklist, known as “Kihon Checklist,”^11^ has already been validated for assessing frailty^12^ and is commonly used nationwide. In addition, physical fitness measurements were recorded and exercise classes were continuously held. To develop social support among local residents, tea‐drinking salons and physical fitness events where villagers can gather were organized. Information regarding these events was disseminated not only via public relations magazines and lectures, but also by informing individuals of the benefits of insurance. Health support services were provided by public health nurses.

Although it is difficult to assess whether each of the aforementioned measures had a direct impact, the following three results were obtained after the measures were implemented: (1) The number of people certified as needing long‐term care in Katsurao Village decreased from 155 in FY 2018 to 136 (87.7%) in FY 2021, (2) the total cost of long‐term care benefits borne by the Katsurao Village decreased; compared to FY 2018, the cost decreased from 244 million yen to 224 million yen (92.0%) in FY 2021, and (3) the monthly long‐term care insurance premiums borne by residents in Katsurao Village decreased from 9800 yen in 2018 to 8200 yen (83.7%) in 2021, down from No. 1 to No. 3 in Japan.

## 3 DISCUSSION

This review reveals that issues related to long‐term care in the aftermath of the Fukushima Daiichi Nuclear Power Plant accident are extremely diverse. In Katsurao Village, the number of older adults requiring light long‐term care increased due to factors such as inactivity in daily life and separation from family and caregivers as a result of the evacuation following the nuclear accident. The physical function of older local residents in affected areas in terms of their muscle strength and balance was also found to be lower among those who were evacuated.^8^, ^13^ In addition, Moriyama et al.^14^ reported that the utilization of long‐term care increased after the disaster, especially among male older adults living alone, without family support. Therefore, it is necessary to improve the healthy life expectancy of these adults through self‐care by focusing on care prevention services for older adults in the affected areas and encouraging them to engage voluntarily in care prevention.

Regarding long‐term care insurance premiums, an increase in the number of older people requiring long‐term care and a decrease in the number of residents who pay the premium was found to affect its increase. Morita et al.^15^ showed that the cost of care in the disaster area increased with the use of light care, and Hasegawa et al.^6^ also showed a similar increase, especially in the evacuation area after the earthquake. Kobashi et al.^16^ revealed that older adults comprise the majority of the returning population, particularly between the age of 65 and 75 years. This suggests the need for manpower in caring for older adults. Therefore, decision makers in the affected areas must prepare to hire a larger workforce to meet the increasing demand for long‐term care services.

Individuals' long‐term care costs are associated with their physical function, as has been revealed in previous studies.^17^, ^18^ Since the immediate reduction of long‐term premiums is impossible, measures to prevent the requirement of long‐term care among the older adults who returned to Katsurao Village should be prioritized by maintaining their physical function. Furthermore, because the prevention services of long‐term care insurance were found to be effective among subjects aged 85 or older whose disability level was low (support level 1),^19^ decision makers should identify a specific group for which prevention services will be more effective.

## 4 CONCLUSION

Following the nuclear accident, several areas faced challenges, such as a large number of local residents with deteriorated health, shortage of manpower to engage in prevention services, and separation of local residents, which made it hard to provide efficient service. Thus, establishing a support scheme with external specialists and researchers following a disaster is necessary to deal with long‐term care issues in each municipality.

## AUTHOR CONTRIBUTION

TZ and NM drafted the manuscript. NI, TA, TM, and YN helped to revise the manuscript. YN, and MT supervised this study. All authors read and approved the final manuscript.

## FUNDING INFORMATION

This study is supported by the Research project on the Health Effects of Radiation organized by Ministry of the Environment, Japan.

## CONFLICT OF INTEREST

None.

## CONSENT

Written informed consent was obtained from the office staff of the Katsurao village to publish this report in accordance with the journal's patient consent policy.

## Data Availability

None.
